# Signaling Network of Forkhead Family of Transcription Factors (FOXO) in Dietary Restriction

**DOI:** 10.3390/cells9010100

**Published:** 2019-12-31

**Authors:** Yizhou Jiang, Fengxia Yan, Zhongping Feng, Philip Lazarovici, Wenhua Zheng

**Affiliations:** 1 Centre of Reproduction, Development & Aging and Institute of Translation Medicine, Faculty of Health Sciences, University of Macau, Macau 999078, China; yb77642@connect.um.edu.mo; 2 Department of Biology, Southern University of Science and Technology, Shenzhen 518055, China; 3 School of Medical Science, Jinan University, Guangzhou 510080, China; yanfengxia0807@163.com; 4 Department of Physiology, Faculty of Medicine, University of Toronto, Toronto, ON M5S 1A8, Canada; zp.feng@utoronto.ca; 5 School of Pharmacy Institute for Drug Research, Faculty of Medicine, The Hebrew University of Jerusalem, Jerusalem 91120, Israel; philipl@ekmd.huji.ac.il

**Keywords:** FOXO, dietary restriction, insulin/IGF-1 signaling pathway, AMPK, sirtuins, mTOR, longevity, calorie restriction mimetics

## Abstract

Dietary restriction (DR), which is defined as a reduction of particular or total nutrient intake without causing malnutrition, has been proved to be a robust way to extend both lifespan and health-span in various species from yeast to mammal. However, the molecular mechanisms by which DR confers benefits on longevity were not yet fully elucidated. The forkhead box O transcription factors (FOXOs), identified as downstream regulators of the insulin/IGF-1 signaling pathway, control the expression of many genes regulating crucial biological processes such as metabolic homeostasis, redox balance, stress response and cell viability and proliferation. The activity of FOXOs is also mediated by AMP-activated protein kinase (AMPK), sirtuins and the mammalian target of rapamycin (mTOR). Therefore, the FOXO-related pathways form a complex network critical for coordinating a response to environmental fluctuations in order to maintain cellular homeostasis and to support physiological aging. In this review, we will focus on the role of FOXOs in different DR interventions. As different DR regimens or calorie (energy) restriction mimetics (CRMs) can elicit both distinct and overlapped DR-related signaling pathways, the benefits of DR may be maximized by combining diverse forms of interventions. In addition, a better understanding of the precise role of FOXOs in different mechanistic aspects of DR response would provide clear cellular and molecular insights on DR-induced increase of lifespan and health-span.

## 1. Dietary Restriction and FOXOs

Dietary restriction (DR), which is defined as a reduction of particular or total nutrient intake without causing malnutrition, has been proved to be a robust method to decelerate the aging process. Besides extended lifespan, animals subjected to DR also display other phenotypes including increased stress resistance, increased insulin sensitivity, improved mitochondrial health, decreased lipid accumulation and reduced body size. For example, mice under DR survive better than ad libitum fed animals under paraquat-induced oxidative stress [1]. Both short- and long-term DR mitigate ischemia reperfusion injury in the brain, heart, liver, and kidney by activating autophagy, reducing the inflammatory response and enhancing antioxidant activity [2,3,4,5,6]. In humans, DR improves several biomarkers of longevity and reduces the risk of developing diabetes, cardiovascular disease and cancer [7,8].

Forkhead box O transcription factors (FOXO) transcription factors have long been considered as a key regulator of aging. The mammalian FOXO family includes four proteins: FOXO1, FOXO3 (or FOXO3a), FOXO4 and FOXO6 [9]. Invertebrates express a single FOXO gene, for example, *daf* (abnormal dauer formation)*-16* in *Caenorhabditis elegans* [10] and *dFOXO* in *Drosophila* [11]. Albeit, FOXOs have distinct expression patterns and functions, they recognize the same consensus sequence TTGTTTAC [12]. In response to changing environment or internal stimuli, FOXOs bind to the promoter of many target genes that are involved in a variety of crucial biological processes such as stress resistance, metabolism, proliferation, cell cycle arrest and autophagy [13,14,15]. FOXOs have been shown to play a key role in DR response. In yeasts, DR failed to extend the chronological lifespan of mutant with deletion of both *FKH1* and *FKH2*, two Forkhead box orthologs which are considered to be evolutionarily conserved with higher eukaryotic FOXO proteins [16]. In rat skeletal muscles, every other day feeding resulted in significantly elevated gene expression of FoxO1 and 4, whereas FoxO3 levels were not statistically significantly elevated [17]. In mice, DR up-regulated mRNA expression levels of FoxO1 in the liver and skeletal muscle, increased the levels of FoxO4 mRNA in the skeletal muscle and adipose tissue, whereas no effect was observed in Foxo3 mRNA levels in these tissues [18]. In addition to the direct DR-induced increase in FOXO proteins expression, DR also alters expression a variety of FOXO target genes, such as heme oxygenase-1 (HO-1), growth arrest and DNA-damage-inducible, alpha (GADD45a), manganese superoxide dismutase (MnSOD), catalase, cyclin-dependent kinase inhibitor 1A (p21) and cyclin-dependent kinase inhibitor 1B (p27) [4,18,19,20,21]. DR regimen did not extend the lifespan of both Foxo3-knockout heterozygous (+/−) and homozygous (−/−) mice, suggesting that FoxO3 is required for DR to extend lifespan [22]. The effect of DR on reducing incidence of tumor was abolished in FoxO1 knockout heterozygous (HT) mice, indicating that FoxO1 is involved in the anti-tumor effects of DR [18]. It was also reported that FoxO1-mediated antioxidant activity and autophagy are essential for the protective effect of DR against liver injury [4]. Moreover, a recent study reveals that FoxO1 also regulate the anti-inflammatory action of DR by interacting with PGC-1α (peroxisome proliferator-activated receptor gamma (PPAR-γ) coactivator 1-alpha) [23]. Interestingly, it was recently reported that in *C. elegans*, daf-16 could modulate immunity and promote longevity by inhibiting feeding behavior, which appears to mimic a DR-like situation [24]. The mechanism by which daf-16 regulates food intake is an interesting question deserving further investigation.

## 2. Signaling Networks of FOXO in DR: Insulin/IGF-1 Signaling Pathway

The activity of FOXOs is negatively regulated by IGF-1 (insulin-like growth factor 1) receptors. Briefly, ligand activation of IGF-1 receptor triggers the phosphorylation of PI3K (phosphoinositide 3-kinase) leading to activation of Akt (also called protein kinase B or PKB) by phosphorylation [25,26]. Akt phosphorylates FOXO proteins and promotes their binding to 14-3-3 proteins and nuclear exclusion [27,28]. FOXO protein levels in the cytosol are regulated by ubiquitin proteasome pathway (UPP) [29].

The insulin/IGF-1 signaling pathway is the first identified, longevity-regulating pathway. In *C. elegans*, mutation in daf-2, mammalian insulin/IGF-1 receptor homolog, results in a doubled lifespan [30]. Worm mutants carrying mutation in *age-1*, the *C. elegans* homolog of the *PI3K*, are also long-lived [31]. daf-16 is required for the extended lifespan of these mutants [32]. Interestingly, the extended lifespan of *eat-2* mutant is not dependent on daf-16 and *eat-2*; *daf-2* double mutants live longer than single mutants [33,34]. These findings suggest that DR and insulin/IGF-1 pathway differently act to extend lifespan in *C. elegans*. In the fruit fly *Drosophila*, DR did not further extend lifespan of a long-lived dwarf *chico* gene mutant, which carries a mutation in the insulin receptor (InR) substrate [35,36]. Conversely, another study found that although insulin-like peptide-5 (*ilp5*) was suppressed by DR, it was not required for DR to extend lifespan [37]. In addition, overexpression of *dfoxo* gene in the fat body increased the sensitivity to DR, but DR could still successfully extend lifespan in both *dfoxo* null mutant and wild-type. These findings indicate that although *dfoxo* modulates in part DR-induced lifespan extension, DR is utilizing additional signaling systems to regulate this process [37,38].

Although the Insulin/IGF-1 signaling pathway is a highly conserved from yeast to human, some major differences in its activity have been found in different species. For example, in mammals, but not in invertebrates, the activity of the insulin/IGF-1 pathway is regulated by growth hormone (GH), a peptide secreted by pituitary [39,40]. GH secretion declines with aging and its decreased secretion parallels the decrease in IGF-1 levels [39]. IGF-1 signaling is involved in the regulation of cell proliferation, stress resistance, apoptosis and tumorigenesis, and decreased IGF-1 level is proved to be lifespan beneficial. Studies in dogs have proved that animals with lower circulating IGF-1 levels tend to have smaller body size and longer lifespan [41,42]. A basic aging theory claims that GH decreases lifespan and that lifespan benefits of DR regimens are attributed to reduced growth stimulation [43,44]. DR has been shown to reduce IGF-1 levels in mice [45]. However, the effect of DR on IGF-1 signaling in humans appears to be more complex. A recent study showed that DR significantly decreased insulin and IGF-1 levels in humans, consistent with observations in rodents [46]. However, DR failed to decrease GH and IGF-1 levels in non-obese men and women [47]. Interestingly, another study demonstrated that although DR without malnutrition did not change IGF-1 levels in humans, a significant reduction was observed when protein intake was restricted [48]. These findings suggest that DR responses are different in rodents and humans, and therefore further studies are required to clarify the physiological and pathological metabolic effects of protein restriction.

Interventions to disrupt the GH/IGF-1 (somatotropic) axis have been shown to have pro-longevity effects in mice. For example, mice with deletion of insulin receptor substrate 1 (IRS1) are long-lived with delay age-related processes [49]. Knockout of the mouse growth hormone receptor/binding protein (GHR/BP) gene also results in an extended lifespan [44,50,51]. Interestingly, lifespan extension by suppression of the GH/IGF-1 system appears to require the complete absence of GH and IGF-1 signaling. It was reported that a single substitution of glycine with lysine at position 119 in bGH (bovine growth hormone) results in the production of a mutated protein that could act as a functional antagonist of GH [52]. Mice carrying such mutation have markedly reduced, low level of GH/IGF-1 signaling, and importantly, a normal lifespan [53]. GHR/BP ^−/−^ knock out mice also displayed DR-like phenotype such as GH resistance, decreased IGF-I levels and reduced body size [44,50,51]. In addition, DR did not alter metabolic parameters including oxygen consumption and respiratory quotient and failed to further extend the lifespan of GH receptor-deficient (GHRKO) mice, indicating that DR and reduced IGF-1/insulin signaling may act through the same mechanism to promote mice longevity [54,55]. Another study demonstrated that wild type mice subjected to DR and GHRKO mice have substantially different expression levels of genes related to insulin signaling, and this difference is tissue-specific [54]. Taken together, the effects of DR and reduced IGF-1/insulin signaling are similar but not identical, and this similarity could differ across species.

## 3. DR and AMPK

The AMP-activated protein kinase (AMPK) is an energy sensor that responds to cellular energy status and mediates metabolism. AMPK is activated by a high AMP/ATP ratio and therefore serves as a sensor of cellular “fuel deficiency”. AMPK activation is proposed to mediate some of the health-protective effects of long-term calorie restriction. AMP binds to AMPK and enables its activation by phosphorylation at Threonine (Thr)-172 by upstream kinases, including liver kinase B1 (LKB1) and calmodulin-dependent protein kinase (CaMKK) [56]. AMPK has been shown to directly regulate activity of FOXOs by phosphorylation [57]. AMPK phosphorylates FoxO3 at sites different from that was previously shown to be regulated by AKT [57]. Interestingly, AMPK appears to increase FoxO3 transcriptional activity without affecting its subcellular localization [57].

In *C. elegans*, aak-2, a homolog of the α-catalytic subunits of mammalian AMPK, regulates longevity in response to increased AMP/ATP ratio or decreased insulin-like signaling [58]. As reduced calorie intake has been shown to increase AMP/ATP ratio, it was hypothesized that aak-2 may mediate DR-induced lifespan extension [59]. Indeed, aak-2/eat-2 double mutant lives as long as eat-2 mutant, indicating that eat-2 deficiency-induced DR may bypass aak-2 to extend lifespan [60]. By contrary, a DR regimen in worms, called sDR, extended lifespan in an aak-2-dependent manner [61]. Worms expressing constitutively active AMPK exhibit increased resistance to oxidative stress, increased daf-16-dependent SOD-3 (Superoxide Dismutase 3) expression and prolonged lifespan [61]. In addition, daf-16 is necessary for both sDR and AMPK activation to extend lifespan and delay age-dependent fitness decline while daf-2 is not required [61]. Therefore, the AMPK/FOXO signaling pathway plays a key role in sDR-mediated lifespan extension. Another study indicated that AMPK and FOXO are necessary for lifespan extension induced by dilution of peptone, while are dispensable for longevity induced by bDR (a DR method achieved by diluting bacteria in liquid culture) and eat-2 (ad1116) mutation [62]. These findings propose that the role of the AMPK/FOXO pathway in DR could be different, depending on various DR regimens [63].

## 4. DR and SIRTUIN

The nicotinamide adenine dinucleotide (NAD)-dependent histone deacetylase silent information regulator 2 (Sir2) that targets both histone and non-histone proteins, including transcription factors involved in diverse processes such as stress resistance, cell differentiation, metabolism and aging is a metabolic regulator conserved across species [64,65]. It was reported that overexpressing Sir2 increases lifespan in both *C. elegans* and *Drosophila* [66,67]. In *C. elegans*, daf-16 is required for the lifespan extension of the sir-2.1 transgene, suggesting a possible interaction between Sir2 and daf-16 [66]. The transcriptional activity FOXO is regulated by reversible acetylation and deacetylation [68]. In response to oxidative stress stimuli, the cAMP response element-binding protein (CREB)-binding protein (CBP) acetylates FOXO and suppresses its transcriptional activity, by attenuating its DNA-binding ability and enhancing phosphorylation by Akt [68,69,70]. Sir2 binds FOXO and deacetylates it to promote FOXO-dependent gene transcription, including genes involved in oxidative stress resistance and glucose production [71]. Sir2 plays a key role in DR-mediated longevity. Animals subjecting to DR showed an increase in Sir2 protein levels [67,72,73]. In yeast, mutations in *dSir2* or *NPT1*, the genes encoding NAD synthesis’ enzymes, blocked DR-induced increased longevity [74]. In *Drosophila*, DR failed to extend lifespan when *dSir2* has no function or severely reduced function [67]. In *C. elegans*, SIR-2.1 binds DAF-16 in a 14-3-3-dependent manner to induce the transcriptional activation of DAF-16 [75]. The lifespan-extending effect in overexpressing sir-2.1 and eat-2 genes is not additive [76]. In addition, eat-2 mediated lifespan extension was suppressed in sir-2.1/eat-2 double mutant [77]. Therefore, sir-2.1 acts similarly to eat-2, to extend lifespan. However the regulation of life span is probably more complex since several other studies suggested that lifespan extension in response to DR can be independent of Sir2 [78,79].

Aging is associated with diminished SIRT1 expression and activity [80,81]. In mammals, DR induces the SIRT1, the closest homolog of *C. elegans* sir-2.1 and *Drosophila dSir2*, and promotes cell survival [82]. In animal models, stimulation of SIRT1 (either with agonists or NAD+ precursors) caused extension of the lifespan and protected against many aging-related diseases, including autoimmune diseases, cardiovascular diseases, neurodegenerative disorders and cancer [83,84,85,86,87,88,89]. In addition, DR induced an increase in physical activity was abolished in SIRT knockout mice [90]. Although daf-16 is required for the lifespan extension of the sir-2.1 overexpression [66], genetic analysis showed that the expression pattern of sir-2.1 and daf-16 is only partially overlapping [77]. UNC-13 regulates neurotransmitter release in the nervous system and serves as an upstream of daf-2, and the lifespan of mutants with some unc-13 alleles is more than doubled [77]. Interestingly, mutation in either sir-2.1 or daf-16 only partially suppressed the unc-13 induced lifespan extension in double mutants, whereas a triple mutant with unc-13; daf-16; sir-2.1 displayed a similar lifespan as the wild type, indicating the existence of separate functions for sir-2.1 and daf-16 in lifespan regulation [77]. The sirtuin activator resveratrol, also extends lifespan through a sir-2.1-dependent but daf-16-independent manner [62,91], which further supports the notion that DR and lifespan regulation via sir-2.1 and daf-16 are not in a simple signaling pathway. Taken together, Sir2 promotes longevity and mediates many of the beneficial effects of DR. Although Sir2/FOXO mediate DR response in some conditions, they can be replaceable under other conditions, and may also act in different signaling pathways to regulate DR response and longevity.

## 5. DR and mTOR

The mammalian target of rapamycin (mTOR), which was first identified as the target of rapamycin, is a conserved, nutrient sensing, serine/threonine protein kinase that regulates growth and metabolism in all eukaryotic cells. mTOR functions in two complexes: mTOR complex 1 (mTORC1) and mTOR complex 2 (mTORC2). These two complexes are consisting of both shared and distinct subunits. Both mTORC1 and mTORC2 contains mLST8/GβL and DEPTOR, while mTORC1 also contains PRAS40 and Raptor, and mTORC2-specific components include rapamycin-insensitive component of mTOR (Rictor), mSin1, and Protor-1/2 [40,92]. mTOR complexes regulate growth, metabolism, autophagy cell death and mRNA translation [93,94].

Inhibition of the TOR signaling pathway has been shown to mimic DR and extend lifespan in invertebrates and mammals. In yeast, inhibition of the TOR signaling pathway by gene mutations or pharmacological inhibition by methionine sulfoximine or rapamycin extended the life span [95]. Cells with suppressed TOR signaling displayed DR-like phenotypes such as an increase in accumulation of glycogen and increased stress resistance, indicating that mTOR signaling may mediate DR response [95]. However, there are also reports indicating that DR did not extend the lifespan of cells lacking TOR1, one of two TOR proteins expressed in yeast [96]. In *Drosophila*, inhibition of TOR signaling pathway by genetic manipulations, extended in a nutritional condition-dependent manner the lifespan [97].

In *C. elegans*, TOR deficiency resulted in a more than doubled lifespan [98]. Diminished TOR signaling extends lifespan by promoting autophagy and inhibiting translation, like in yeast [93,99]. Mutation in daf-16 did not abrogate the extended lifespan of LET-363/TOR-(RNAi) worms. However, RNAi directly towards TOR did not further extend the lifespan of long-live daf-2 mutant, suggesting that mTOR may act as a downstream target or independent of daf-16 [98]. daf-15, the *C. elegans* ortholog of Raptor, was shown to interact with let-363 to regulate metabolism and lifespan [100]. A heterozygous daf-15 mutation led to a 30% increase in mean lifespan and increased fat accumulation, and mutation in daf-16 suppressed longevity effect of daf-15, but did not affect fat accumulation in daf-15 mutant [100]. In addition, daf-16 was found to inhibit daf-15 transcription [100]. Therefore, in *C. elegans*, the regulation of let-363/daf-15 signaling on lifespan is dependent on daf-16, whereas its metabolic effect is independent of daf-16.

In mice, late-life treatment with rapamycin resulted in an about 10% increase in lifespan [101]. Another study with early-life intervention of rapamycin indicated similar results [102]. Moreover, mice exposed to a DR diet had lower levels of mTOR activity [103]. These findings suggest that mTOR mediates in part mice lifespan extension in response to DR. A study comparing features of rapamycin-treated mice and DR-treated mice demonstrated that rapamycin treatment resulted in an increase in fasting glucose which was not observed in DR animals, without altering fasting insulin, leptin, IGF-1, T4 or FGF-21, which were significantly changed after a five-month DR regimen [104]. It was also reported that rapamycin and DR have quite different effects on transcriptome and metabolome of mice liver, revealing that a combination of these two interventions may produce more benefits than each individual treatment [105]. In addition, a recent study found that although reduced mTOR signaling and DR extended lifespan in rodents, reduced mTOR signaling solely, it did not decrease aging-related mortality, but increased resilience to mortality rate during the aging process [106]. Such distinct mortality patterns have been suggested to reflect differences in insulin sensitivity, mitochondrial biology, xenobiotic metabolism and fatty acid oxidation [106]. To sum up, DR extends lifespan by suppressing TOR activity in a daf-16/FOXO-dependent manner [9]. The findings based on genetic mTOR manipulations and pharmacological treatments indicate that although diminished mTOR signaling has a vital role in DR response, these two longevity-benefiting interventions may act through both distinct and overlapping signaling pathways.

## 6. Functional Cross Talk Between FOXO-Related Signaling Pathways in DR

The Insulin/IGF-1, AMPK, SIR2, mTOR interact with FOXO to regulate DR response. Besides interacting with FOXO in a single signaling pathway, these signaling modules are serving many interconnected cellular pathways [40]. In the Insulin/IGF pathway, AKT is activated by phosphorylation at Thr-308 by PDK-1, or at Ser-473 by mTORC2 [107]. AKT regulates a variety of downstream effector substrates. AKT can also indirectly activates mTORC1 through TSC1/TSC2 (tuberous sclerosis complex 1/tuberous sclerosis complex 2) and Rheb (Ras homolog enriched in brain) [108]. The activated mTORC1, in turn, inhibits AKT, generating a negative feedback signaling loop [108]. A similar negative feedback mechanism exists between AKT and AMPK. AKT phosphorylates AMPKα1/α2 at Ser-485/491 to attenuate the activation of AMPK by phosphorylation at Thr-172 [109,110]. Reversely, AMPK phosphorylates insulin receptor substrate-1 (IRS-1) at Ser-794, which inhibits PI3K/AKT signaling [111,112]. In addition, AMPK can also upregulate the Akt PH Domain and leucine rich repeat protein phosphatase 2 (PHLPP2) to repress Akt activation [113]. However, on the other hand, AMPK can promote AKT phosphorylation independent of IRS-1 [114,115]. Therefore, the antagonism between AMPK and AKT phosphorylation activities is complex and needs further investigations [116].

It is well-established that AKT and AMPK phosphorylate FOXOs at different amino acids, and result in inactivation or activation, respectively. Interestingly, FOXOs can, in turn, regulate insulin signaling and AMPK activation. Under stress conditions, activated FoxO1 stimulates Akt activation through the suppression of mTORC1 and the elevation of mTORC2 activity [117]. In addition, FOXO can also amplify insulin signaling by upregulating insulin receptor (InsR) mRNA level [118,119]. FOXOs promotes the transcription of Sestrin 3 (a highly conserved, antioxidant, stress-inducible protein that inhibits TORC1 signaling) and led to AMPK activation [120,121].

The interaction between AMPK and Sirtuins is also bidirectional. It was reported that AMPK could indirectly enhance SIRT1 activity by increasing cellular NAD+ levels [122]. On the contrary, SIRT1 deacetylates LKB1 and led to the activation of AMPK [123,124]. SIRT1 was also shown to deacetylate Akt to promote its binding to phosphatidylinositol (3,4,5)-trisphosphate (PIP3) [125]. However, in tumor cells, SIRT1 could also negatively regulate AKT pathway by deacetylation of phosphatase and tensin homolog (PTEN) tumor suppressor protein [126]. In addition, another member of the mammalian Sirtuin family SIRT6 was shown to suppress the expression of Akt signaling-related genes [125]. Therefore, Sirtuins appears to have dual effects on AKT signaling.

The mTOR signaling is also regulated by AMPK and Sirtuins. mTORC1 is negatively regulated by both AMPK and Sirtuins. AMPK can directly phosphorylate tuberous sclerosis complex 2 (TSC2), also known as Tuberin, and Raptor to suppress mTORC1 [127]. mTORC1 is also negatively regulated by SIRT1, while SIRT1 modulates mTORC2 by a SIRT1/mTORC2/Akt signaling pathway [40,128,129]. mTORC2 inhibits the transcriptional activity of FOXO by inducing Akt hyper-activation [130]. FOXO could inhibit mTORC1 activity through activation of TSC1 and increasing mTORC2 activity by elevating Rictor [117,131].

The interactions between FOXOs and co-activators, for example, PGC-1α, also play important roles in DR response [23]. PGC-1α has been shown to interact directly with both FoxO1 and FoxO3 and activate FOXO-dependent gene transcription [132,133]. In addition, FOXOs can bind and stimulate the PGC-1α promoter, representing an auto-regulatory feedback mechanism [133,134]. Also, SIRT1 can interact with and deacetylate PGC-1α to enhance its transcriptional activity [135].

## 7. Conclusions and Perspectives

**Figure 1:**
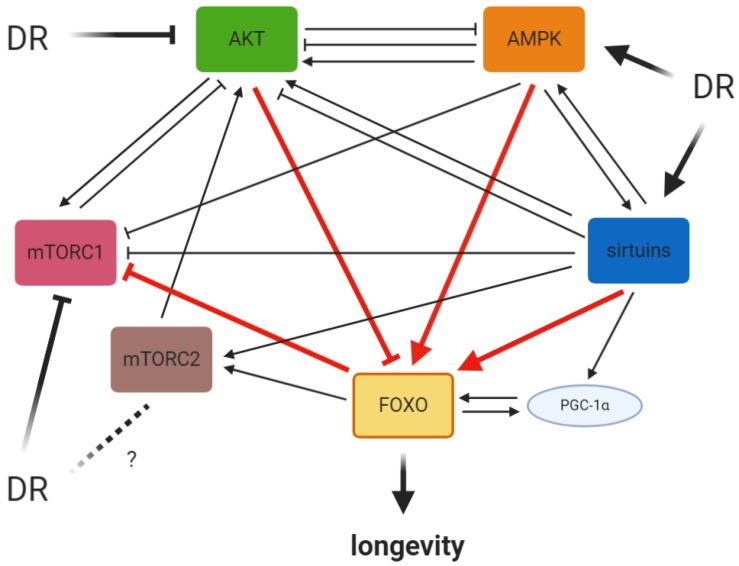
The signaling network of forkhead box O transcription factors (FOXO)-related pathways in dietary restriction (DR).

The network of DR-related signaling pathways is highly interconnected enabling signaling cross-talk (Figure 1). Although different DR regimens have been shown to lead to the same endpoint, they utilize distinct and overlapped signaling modules to extend lifespan and health-span. Although FOXOs are required for longevity induced by many different DR regimens, it only partially mediates the DR response. One possible hypothesis of such diversity is claiming that different nutrients in the various DR regimens differentially stimulate energy-sensing pathways [62,63]. For example, reduced carbohydrate intake prefers activation of AMPK/FOXO pathway, whereas TOR signaling may mediate responses to restricted amino acid intake [62,136]. Interestingly, an important study in humans demonstrated that diminished protein, but not calorie intake, could decrease circulating IGF-1 levels, suggesting that protein food supply may be a key determinant of the effect of DR interventions [48]. As many studies are focusing on DR methods without malnutrition, further studies aimed to define the specific role of different dietary nutrients in DR are needed. In addition, a better understanding of the precise role of FOXOs in different aspects of DR response would provide a clear insight as to how DR confers lifespan and health-span benefits.

Despite DR has a robust positive impact on several parameters of health and reduces the risk of developing age-related diseases in humans, whether or not DR could extend lifespan in humans remains to be clarified. In addition, DR regimens may be difficult to be implemented and managed in our daily life. Therefore, eliciting DR-related genetic pathways using drugs could be a promising option to achieve DR-like benefits. These potential new drugs defined as calorie restriction mimetics (CRM) could simulate beneficial effects of DR, without limiting food intake. For example, metformin, a biguanide drug commonly used to treat type-2 diabetes, has been shown to extend lifespan and delay the aging process in a variety of species including silkworm *Bombyx mori*, *C. elegans*, mice and rats [137,138,139,140]. Although metformin is a potent AMPK activator in all these species, its beneficial action varied. In silkworm *B. mori*, metformin extends lifespan through AMPK-p53-FOXO pathway [140]. In *C. elegans* and mice, the longevity-promoting effect of metformin was attributed to the activation of AMPK/Nrf2 pathway [137,139]. Surprisingly, metformin failed to extend lifespan in *Drosophila* although it activates AMPK and reduces lipid accumulation [141]. Resveratrol, a natural polyphenol antioxidant, has been found to extend lifespan in *C. elegans*, *Drosophila*, zebrafish, mice and rats [91,142,143,144]. The longevity benefits of resveratrol are dependent on sirtuins, FOXOs and AMPK [124,142,145]. Several studies demonstrated that resveratrol increased longevity only in obese in mice [146,147]. Rapamycin, an antibiotic targeting mTOR, is also a promising CRM in a variety of organisms [95,101]. It is important to stress that rapamycin could cause adverse effects including diabetic-like symptoms [148]. The precise pharmacological action of these CRMs on DR-related or -independent signaling pathways remains to be further investigated. More efforts should be made in research and development of novel safe CRMs with minimal adverse effects.

In conclusion, the present review summarizes recent research advances in unveiling the molecular mechanisms of FOXOs and related signaling pathways contributions to DR response. Additional experimentation is required to establish a better understanding of the complex signaling network which can be targeted in developing new strategies to extend human longevity. As different DR regimens or DR mimetics can elicit both distinct and overlapping DR-related signaling pathways and may have other actions beyond DR-related mechanisms, it is tempting to propose that the longevity benefits of DR may be maximized by combining diverse forms of DR interventions.
